# Purple corn extract improves benign prostatic hyperplasia by inhibiting 5 alpha-reductase type 2 and inflammation in testosterone propionate-induced rats

**DOI:** 10.3389/fphar.2024.1485072

**Published:** 2025-01-03

**Authors:** Sung-Ok Kim, Arin Choi, Hee-Hwan Lee, Jae-Yong Lee, Sang Jae Park, Byung-Hak Kim

**Affiliations:** Medience Co., Ltd., Chuncheon, Republic of Korea

**Keywords:** purple corn extract, anthocyanin, benign prostatic hyperplasia, 5α-reductase type 2, androgen receptor, inflammation

## Abstract

Benign prostatic hyperplasia (BPH) is a health issue caused by an enlarged prostate in older men. Its prevalence increases with age, and it results in urination-related problems. This works studied the effect of purple corn extract (PCE) on improving BPH symptoms using a testosterone propionate (TP)-induced rat model. PCE reduced the enlargement and weight of the prostate through the inhibition of the conversion of testosterone to dihydrotestosterone (DHT) by inhibiting 5α-reductase type 2 (*Srd5a2*) in TP-induced BPH rats. In these rats, PCE decreased androgen receptor (AR) expression, AR-mediated signaling, and cell proliferation and increased apoptotic cell death. Finally, PCE exhibited anti-inflammatory activity through the regulation of the nuclear factor-kappa B (NF-κB) and nuclear factor erythroid 2-related factor 2 (Nrf-2) axis. These results indicate that the *Srd5a2* inhibition and anti-inflammatory activity are some of the beneficial effects of PCE that improve BPH symptoms.

## 1 Introduction

Purple corn (Zea mays L.) is a group of flint maize varieties (Zea mays indurata) originating in the Andean region of South America and is commonly cultivated in the Andes of Peru, Bolivia, Ecuador, and Argentina. Typically, it contains a high contents of purple color pigments in the cob and pericarp, and these pigments are widely used to prepare colorants for traditional foods and beverages (Luna-Vital et al., 2017; Chatham et al., 2020). Recently, purple corn has been extensively utilized in the novel ingredient markets and pharmaceutical industries because it contains many bioactive phenolic compounds such as anthocyanins, phenolic acids, and flavonoids, which are beneficial for health (Lao et al., 2017; Cristianini and Guillén Sánchez, 2020; Kim et al., 2023). Anthocyanins are major components and are responsible for the purple reddish-purple color of purple corn. They are a class of water-soluble flavonoids composed of a basic C6-C3-C6 skeleton and are glycosylated polyhydroxy and/or polymethoxy derivatives of 2-phenylbenzopyrilium (Smeriglio et al., 2016). Purple corn anthocyanins are mainly composed of one or more glycosylated sugar molecules in anthocyanidins, where the sugar molecules are glucosides and malonyl or dimalonyl glucosides and the anthocyanidins are cyanidin, pelargonidin, and peonidin (Chatham and Juvik, 2021; Kim et al., 2023).

Benign prostatic hyperplasia (BPH) is a common representative gender-dependent urological disorder in the older male population (Lim, 2017; GBD, 2019 Benign Prostatic Hyperplasia Collaborators, 2022). It occurs due to progressive benign enlargement of the prostate tissue mediated by hyperproliferation of epithelial and stromal cells in the prostate transition zone, leading to compression of the urethra and development of bladder outlet obstruction (BOO) (Prajapati et al., 2013; Hata et al., 2023). BPH causes many problems in the lower urinary tract, and its prevalence increases with age. It starts at 40–45 years of age, reaches 50%–60% in men in their 60s, and reaches up to 80%–90% in men over 70 (Ng and Baradhi, 2022), indicating the need to develop novel agents to effectively and safely treat BPH in middle-aged and elderly adults. However, the etiology and molecular mechanisms of BPH are not fully understood despite being studied extensively, but several risk factors that trigger BPH have been hypothesized.

Prostatic inflammation is considered to be a risk factors for the pathogenesis of BPH. The inflammatory response is triggered by several factors, and the association between BPH and prostatic inflammation has been demonstrated in studies using BPH specimens and prostate tissue-derived cell lines (Robert et al., 2009; Gandaglia et al., 2013; Tsunemori and Sugimoto, 2021). Autoimmune responses against self-antigens released from injured tissues play an important role in cellular hyperproliferation during inflammation. A representative self-antigen is prostate-specific antigen (PSA), which is the most important candidate for activating CD4^+^ T cells in patients with granulomatous prostatitis (Robert et al., 2009). The relationship between BHP and inflammation has been further demonstrated using a variety of molecular and cellular biology techniques in prostate epithelial cells and stromal fibroblasts (Begley et al., 2008; Bardan et al., 2014; Madersbacher et al., 2019). These cells produce various types of proinflammatory mediators, including cytokines, enzymes, and chemokines, and these mediators are sufficient to induce hyperproliferation of prostate epithelial and stromal cells. In fact, studies in patients with BPH have shown lower expression of proinflammatory mediators with clinical agents than with placebos (Roehrborn, 2008; Vela Navarrete et al., 2003). Regardless of the initial triggers, proinflammatory mediators exacerbate BPH symptoms through inflammation and proliferation of irregular epithelial and stromal cells in the prostate (De Nunzio et al., 2016).

Many studies suggest the importance of inflammation in the development of BPH, indicating that anti-inflammatory agents may effectively treat this condition. Purple corn contains a large amount of health functional materials such as anthocyanins, phenolic acids, and flavonoids, thus, it has recently become an emerging star in the novel ingredients market and the pharmaceutical industry. Therefore, we suggested that PCE could be effectively used to treat BPH through the health benefit functions of purple corn, including anti-inflammatory activity. The present study aimed to investigate the anti-inflammatory activity of purple corn extract (PCE) in rats using a BPH model. PCE was prepared from the husks and cobs of purple corn, and BPH was induced in rats via subcutaneous injection of testosterone propionate (TP) in rats. We found that PCE is a promising therapeutic agent that can ameliorate BPH symptoms through the regulation of AR signaling and anti-inflammatory activity.

## 2 Materials and methods

### 2.1 Plant materials and PCE preparation

The purple corn was developed and registered by Gangwon Agricultural Research and Extension Services (Chuncheon, Republic of Korea), and PCE was prepared from a mixture of the dried husks and cobs of the purple corn, as previously described (Kim et al., 2022; Lee et al., 2023).

### 2.2 Chemicals and reagents

Testosterone propionate (TP) and finasteride were purchased from Wako Pure Chemicals Industries, Ltd. (Tokyo, Japan) and Glentham Life Sciences Ltd. (Corsham, Wiltshire, UK), respectively. Primary antibodies specific to PCNA (#13110), cyclin D1 (#2978), COX-2 (#1228), and NF-κB p65 (#8242), and a SignalStain^®^ Boost IHC Detection Reagent (#8114 and #8125), and a SignalStain^®^ DAB Substrate Kit (#8059) were purchased from Cell Signaling Technology (Danver, MA, USA). Antibodies specific to IL-6 (MA5-45070), TNF-α (PA1-40281), and NRF-2 (PA1-38312) were obtained from Invitrogen (Waltham, MA, USA), and AR (ab133273), PSA (A01505-1), SRC-1 (sc-32789), and caspase-3 (NB100-56113) were purchased from Abcam (Cambridge, UK), Boster Biological Technology (Pleasanton, CA, USA), Santa Cruz Biotechnology (Santa Cruz, CA, USA), and Novus Biologicals (Centennial, CO, USA), respectively. Mayer’s hematoxylin solution (S3309) and a One-Step Green Fluorescence TUNEL Apoptosis Detection Kit (KTA2010) were obtained from Dako/Agilent (Santa Clara, CA, USA) and Abbkine (Atlanta, GA, USA), respectively.

### 2.3 Cell culture and viability assay

Human prostate adenocarcinoma LNCaP (CRL-1740) cells, which are sensitive to both androgens and estrogen, were obtained from the American Type Culture Collection (Manassas, VA, USA) and cultured in RPMI supplemented with 10% fetal bovine serum (FBS, HyClone, Logan, UT, USA) and antibiotics (Welgene, Gyeongsan, Korea). To determine cell viability, the cells were seeded at a density of 1 × 10^4^ cells/well in a 96-well plate containing fresh medium. After one night, the cells were incubated with the vehicle alone, with different concentrations of PCE or TP, or with different concentrations of PCE in the presence of 100 nM TP for 24 h. Cell viability was determined at 450 nm using a microplate reader (Molecular Devices, Sunnyvale, CA, USA) after further incubation for 2–4 h at 37°C following the addition of EZ-CyTox Enhanced Cell Viability Assay Reagent (Daeil Lab Service, Seoul, Korea).

### 2.4 Animal experiment

Eight-week-old male Wistar rats (200 ± 30 g) were supplied by Dae Han Bio Link Co., Ltd. (Eumseong, Korea), and housed in the laboratory animal center of EBO (Cheongju, Republic of Korea) with a controlled room temperature (20–25°C and 40%–60% humidity) and a 12/12 h light/dark cycle. After 1 week of acclimatization, the rats were divided into six groups (*n* = 7) and castrated to eliminate the effects of endogenous testosterone, except those in the normal control group, where a sham operation was performed to provide the same conditions. After 7 days of recovery, the rats were orally administered a vehicle, PCE (2, 10, or 50 mg/kg), or finasteride (10 mg/kg) daily for 28 days. BPH was induced by subcutaneously injecting TP (3 mg/kg) into all rats for 4 weeks except those in the normal control group, as previously reported (Cha et al., 2015). The PCE and finasteride were freshly prepared in distilled water every day before administration, and the TP was prepared with olive oil. All experimental protocols were approved by the Institutional Animal Care and Use Committee (IACUC) of EBO Co., Ltd. (IACUC approval number: EBOA-2017-17).

### 2.5 Enzyme-linked immunosorbent assay (ELISA)

To determine the amounts of testosterone and dihydrotestosterone (DHT), serum was separated via centrifugation from blood samples collected from the abdominal veins of the rats. The serum testosterone and DHT concentrations were determined using commercial ELISA kits for testosterone (ADI-900-065, Enzo Life Sciences, Farmingdale, NY, USA) and DHT (OKEH02531, Aviva Systems Biology, San Diego, CA, USA) according to the manufacturer’s instructions.

### 2.6 Quantitative real-time polymerase chain reaction (qRT-PCR)

Total RNA was isolated from the prostate tissues or LNCaP cells using a RNeasy Mini Kit (Qiagen, Germany) according to the manufacturer’s instructions. Total RNA was quantified by measuring absorbance at 260–280 nm using a spectrophotometer (UV-2450, Shimadzu, Japan), and cDNA was synthesized using a WizScript cDNA Synthesis Kit (Wisbio Solutions, Seongnam, Korea). qRT-PCR was performed using an iTaq™ Universal SYBR One-step kit (Sigma-Aldrich, St. Louis, MO, USA) with a Chromo4 Real-Time PCR Detection System (Bio-Rad Laboratories, Hercules, CA, USA). Comparative C_t_ quantification of the data was performed to derive a value for each sample, which was normalized to the value of the housekeeping gene glyceraldehyde-3-phosphate dehydrogenase (GAPDH). The sequences of the oligonucleotide primers are summarized in Table 1.

**TABLE 1:** Primer sequences used for quantitative real-time PCR.

Target genes		Primer sequences
*Srd5a2*	Forward	5′-GTG​GAG​CAA​ATG​ACA​ACT​TCT​G-3′
	Reverse	5′-GGC​TTC​TGG​TAT​CGC​TGT​ATT-3′
AR	Forward	5′-TAG​CCC​CCT​ACG​GCT​ACA-3′
	Reverse	5′-TTC​CGA​AGA​CGA​CAA​GAT​GGA​C-3′
PSA	Forward	5′-GCC​TCT​CGT​GGC​AGG​GCA​GT-3′
	Reverse	5′-CTG​AGG​GTG​AAC​TTG​GGC​AC-3′
*NFR2*	Forward	5′-TCC​CAA​ACA​AGA​TGC​CTT​GT-3′
	Reverse	5′-AGA​GGC​CAC​ACT​GAC​AGA​GA-3′
*H O -1*	Forward	5′-CAT​CCG​TGC​AGA​GAA​TTC​TG-3′
	Reverse	5′-CTG​GTA​TGG​GCC​CCA​CTG​GC-3′
*IL-1β*	Forward	5′-GCA​CGA​TGC​ACC​TGT​ACG​AT-3′
	Reverse	5′-AGA​CAT​CAC​CAA​GCT​TTT​TTG​CT-3′
*IL-6*	Forward	5′-CAG​GAA​TTG​AAT​GGG​TTT​GC-3′
	Reverse	5′-AAA​CCA​AGG​CAC​AGT​GGA​AC-3′
*IL-8*	Forward	5′-TTT​TGC​CAA​GGA​GTG​CTA​AAG​A-3′
	Reverse	5′-AAC​CCT​CTG​CAC​CCA​GTT​TTC-3′
*IL-12*	Forward	5′-CTT​GTG​GCT​ACC​CTG​GTC​CT-3′
	Reverse	5′-GAG​TTT​GTC​TGG​CCT​TCT​GG-3′
*TNF-α*	Forward	5′-CTG​GGC​AGG​TCT​ACT​TTG​GG-3′
	Reverse	5′-CTG​GAG​GCC​CCA​GTT​TGA​AT-3′
*iNOS*	Forward	5′-GGT​GGA​AGC​GGT​AAC​AAA​GG-3′
	Reverse	5′-TGC​TTG​GTG​GCG​AAG​ATG​A-3′
*COX-2*	Forward	5′-GCC​AAG​CAC​TTT​TGG​TGG​AG-3′
	Reverse	5′-GGG​ACA​GCC​CTT​CAC​GTT​AT-3′
*GAPDH*	Forward	5′-GAC​CAC​AGT​CCA​TGC​CAT​CA-3′
	Reverse	5′-TCC​ACC​ACC​CTG​TTG​CTG​TA-3′

### 2.7 Histological and immunohistochemical analyses

Prostate tissue samples were fixed in a paraformaldehyde solution (4% paraformaldehyde in a 0.5 M phosphate buffer), washed with tap water, dehydrated with graded ethanol, and embedded in paraffin. The paraffin blocks were cut into 4 μm thick sections. The sections were mounted on glass slides, deparaffinized, rehydrated with graded ethanol, and stained with Mayer’s hematoxylin solution for histopathological analysis.

For immunohistochemical analysis, rehydrated slide sections were unmasked with a 10 mM sodium citrate buffer, quenched with endogenous peroxidase in 3% hydrogen peroxide, blocked in PBS containing 10% goat serum, and incubated at 4°C for overnight with the appropriate primary antibodies at dilutions of 1:50-100. The sections were incubated with a SignalStain^®^ Boost IHC Detection Reagent compatible with the primary antibody, subsequently stained with a SignalStain^®^ DAB Substrate Kit, and counterstained with a 0.2% Mayer’s hematoxylin solution. Digital images were obtained using the LAS Microscope Software (Leica Microsystems, Wetzlar, Germany) or BX53T Microscope (Olympus, Japan).

### 2.8 Terminal deoxynucleotidyl transferase dUTP nick-end labeling (TUNEL) staining

The apoptotic cells in the prostate tissues were determined with TUNEL staining using a one-step green fluorescence TUNEL Apoptosis Detection Kit according to the manufacturer’s instructions. The TUNEL-positive cells were counted using an inverted fluorescence microscope (Carl Zeiss, Jena, Germany).

### 2.9 Statistical analysis

Data were represented as means ± standard errors of means (SEMs). Statistical analysis was carried out using GraphPad Prism 5.0 (GraphPad Software, San Diego, CA, USA), and significance was determined using a two-tailed Student’s t–test. Differences were considered statistically significant at *p* < 0.05.

## 3 Results

### 3.1 PCE reduces prostatic enlargement

Hybrid grains of purple corn were developed by the Gangwon Agricultural Research and Extension Services. The purple corn contained large amounts of anthocyanins in the husks, cobs, and leaves. Among them, 10 kinds of anthocyanins were identified, among which the cyanidin family was the most abundant (Li et al., 2008). According to a high-performance liquid chromatography (HPLC) analysis, PCE prepared from a mixture of the dried husks and cobs contained a large amount of cyanidin-3-O-glucoside (C3G), a member of the cyanidin family, and the content was 18.43 ± 0.58 mg/g (Kim et al., 2022; Lee et al., 2023).

**FIGURE 1:**
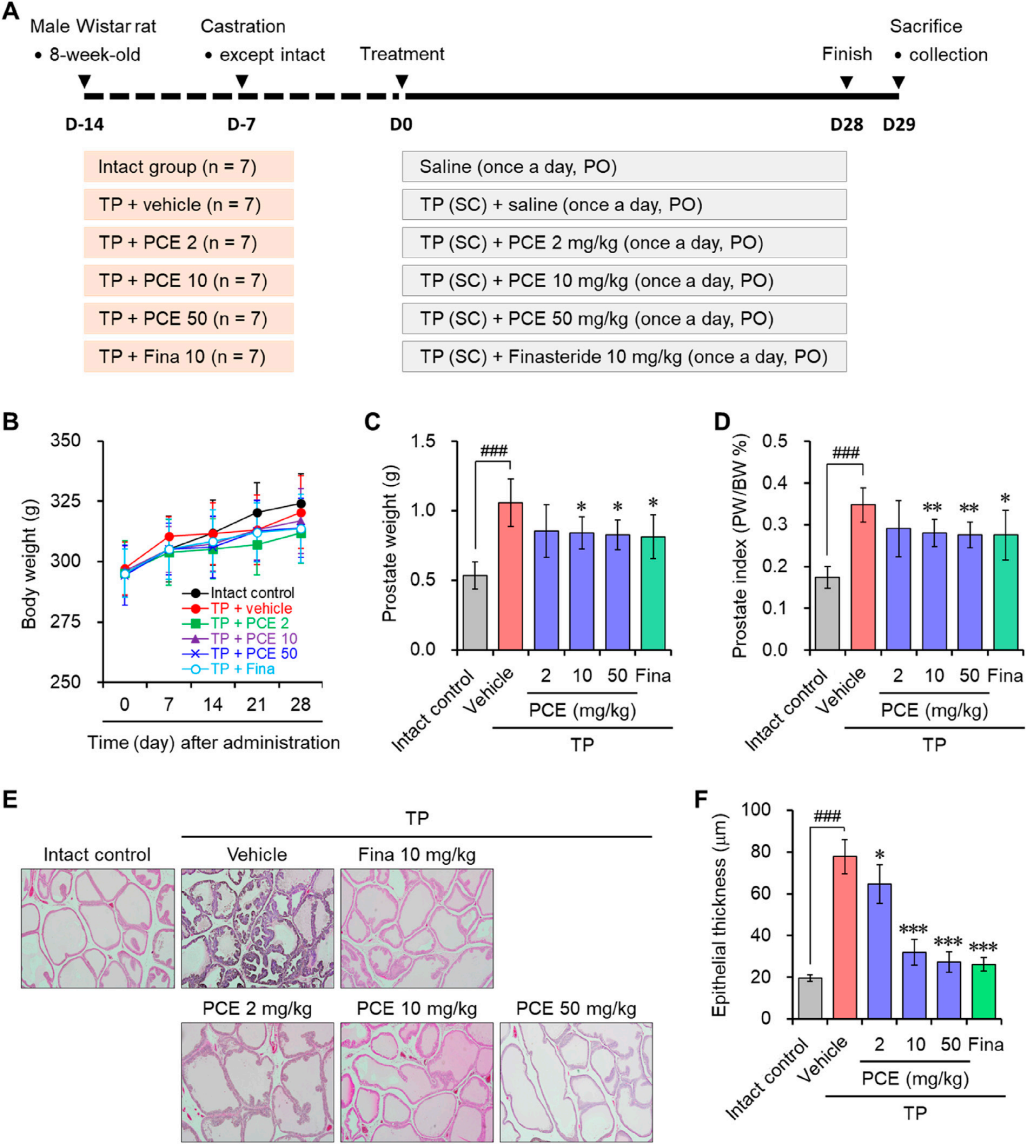
Effect of PCE on prostatic enlargement in rats with TP-induced BPH. **(A)** A schematic diagram of the experimental design. **(B)** The body weights of the rats were measured at 7-day intervals. **(C)** The prostate weights and **(D)** prostate weight to body weight (PW/BW) ratios of the rats were measured at the ends of the experiments. The prostate tissue sections were stained with Mayer’s hematoxylin solution, and **(E)** representative images (original magnification ×10) and **(F)** the epithelial thickness are presented. The results are expressed as means ± SEMs (*n* = 7). ^###^ *p* < 0.0005 versus the normal control group. *p < 0.05, **p < 0.005, and ***p < 0.0005 versus the TP-induced BPH group. Fina, finasteride; TP, testosterone propionate (30 mg/kg).

To investigate the effect of PCE on BPH symptoms, PCE was orally administered to male Wistar rats that had developed BPH due to subcutaneously injections of TP (Figure 1A). Administration of 2–50 mg/kg PCE or 10 mg/kg finasteride did not affect the body weights of the rats compared to the normal and TP-administered control groups during the experimental periods (Figure 1B). On the other hand, the prostate weight was considerably increased in the rats in the TP-induced control group compared to the normal group, and the weight was reduced via administration of PCE or finasteride. The effect of 10 or 50 mg/kg PCE was similar to that of finasteride (Figure 1C). Furthermore, the relative ratio of prostate weight (PW) to body weight (BW) was also significantly elevated in the TP-induced control group compared to the normal group. This ratio was reduced by administration of PCE or finasteride, and these reduction patterns were similar to those of the prostate weight (Figure 1D). These results imply that PCE may inhibit the development and/or progression of BPH induced by TP in rats.

BPH is an enlarged prostate due to an expansion of the prostate and surrounding tissues mediated by hyperproliferation of epithelial and stromal cells. H&E staining was performed to monitor the histological changes in the prostate. Indeed, the staining results indicated that greatly increased hyperplasia of the prostate epithelium was observed in the tissue sections from the TP-induced control group compared to the normal group. In comparison with the TP-induced control group, PCE administration effectively reduced the hyperplasia of the prostate epithelium. In particular, the effects of 10 and 50 mg/kg PCE were similar to that of the finasteride-treated group, which was similar to that of the normal group (Figures 1E, F). These results suggest that the reduction of BPH by PCE may be associated with suppressing the proliferation of cells comprising the prostate.

### 3.2 PCE inhibits the conversion of testosterone to DHT by inhibiting 5α-reductase type 2

Hormonal imbalances, especially increases in the DHT concentration, play an important role in the pathogenesis of BPH. Therefore, regulation of DHT levels is a promising therapeutic method for treating BPH, and several agents are being used in clinical trials to treat BPH by suppressing DHT production. Among these agents, finasteride and dutasteride are currently available agents to treat BPH by reducing DHT. They dramatically reduce prostatic and serum DHT levels while increase serum testosterone levels by targeting 5α-reductase isoenzymes (Roehrborn et al., 1999; Roehrborn et al., 2002; Roehrborn et al., 2003).

**FIGURE 2:**
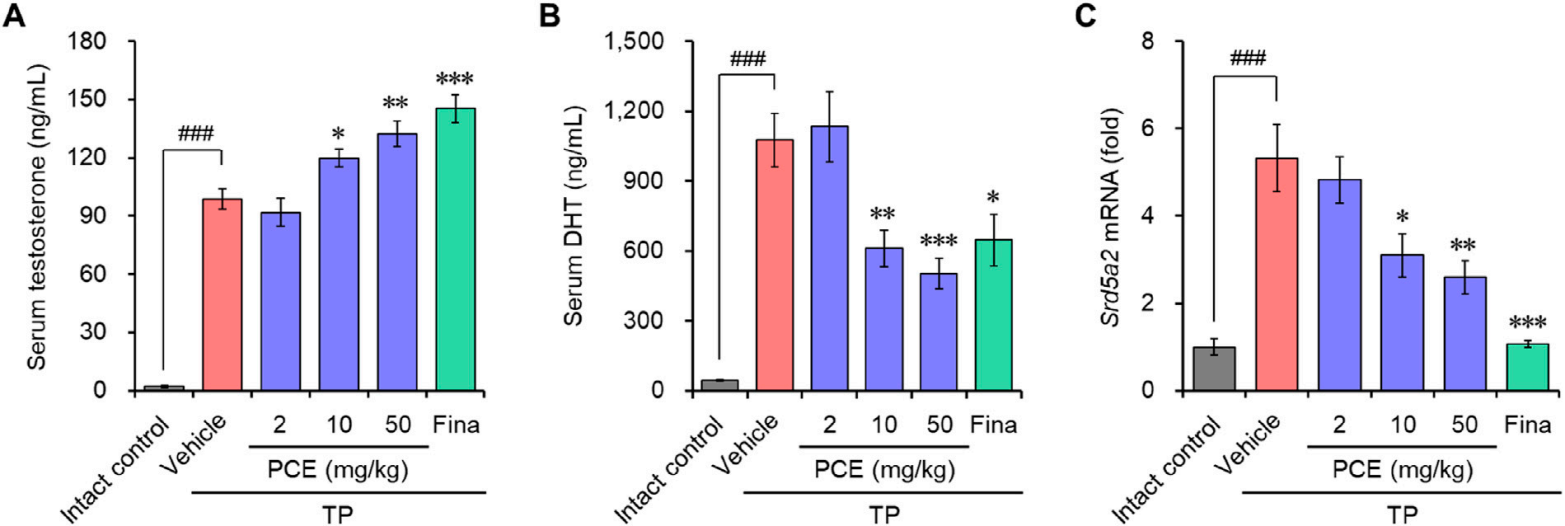
Effect of PCE on serum testosterone and DHT, and mRNA expression of 5α-reductase type 2 (*Srd5a2*) in rats with TP-induced BPH. The concentrations of **(A)** testosterone and **(B)** DHT in serum were determined via ELISA. **(C)** The mRNA expression of 5α-reductase type 2 (*Srd5a2*) was analyzed using quantitative real-time PCR. The results are expressed as relative values compared to the vehicle-treated control and are presented as means ± SEMs (*n* = 3). ^###^ *p* < 0.0005 versus the normal control group. *p < 0.05, **p < 0.005, and ***p < 0.0005 versus the TP-induced BPH group. Fina, finasteride (10 mg/kg); TP, testosterone propionate (30 mg/kg).

Serum testosterone and DHT were quantified to identify whether the anti-BPH activity of PCE was derived from the regulation of these hormonal imbalances. Both testosterone and DHT amounts were dramatically increased in the TP-induced control group compared to the normal group. In particular, PCE administration increased the serum testosterone levels in a concentration-dependent manner, leading to a significant increase compared to the TP-induced control group (Figure 2A). In contrast, the serum DHT levels were decreased concentration-dependent manner by PCE treatment compared to the TP-induced control group, and 10 and 50 mg/kg PCE showed statistically significant differences compared to the TP-induced control group (Figure 2B). Finasteride also regulated the levels of both hormones, which increased serum testosterone while decreasing serum DHT.

High levels of DHT are primarily associated with BPH in people over the age of 50 who were assigned male at birth. Since DHT is converted from testosterone by steroid 5α-reductase isoenzymes (Chislett et al., 2023), the mRNA levels of 5α-reductase type 2 (SRD5A2), one of the isoenzymes, were investigated to determine whether the decrease in DHT due to PCE administration was involved in blocking the expression of this enzyme. Consistent with the increase in DHT levels after TP injection, the expression of *Srd5a2* was markedly elevated in the TP-induced control group but significantly decreased in a concentration-dependent manner in the PCE-administered group (Figure 2C). Interestingly, the decreases in the serum DHT and *Srd5a2* levels and the increases in the serum testosterone levels showed a highly relevant pattern. Finasteride, a well-known 5α-reductase inhibitor, also effectively decreased the expression of *Srd5a2*. These results indicate that one of the modes of action by which PCE reduces BPH symptoms is similar to that of finasteride.

### 3.3 PCE inhibits androgen receptor signaling pathway

The binding of androgen hormones to their receptors initiates androgen receptor (AR) signaling cascades, and activating this signaling plays a critical role in promoting cell growth, BPH, and cancer (Madersbacher et al., 2019; Michmerhuizen et al., 2020). During this signaling cascade, steroid receptor coactivator-1 (SRC-1) participates in androgen-induced AR activation by directly binding to the ARs (Chen et al., 2024).

**FIGURE 3:**
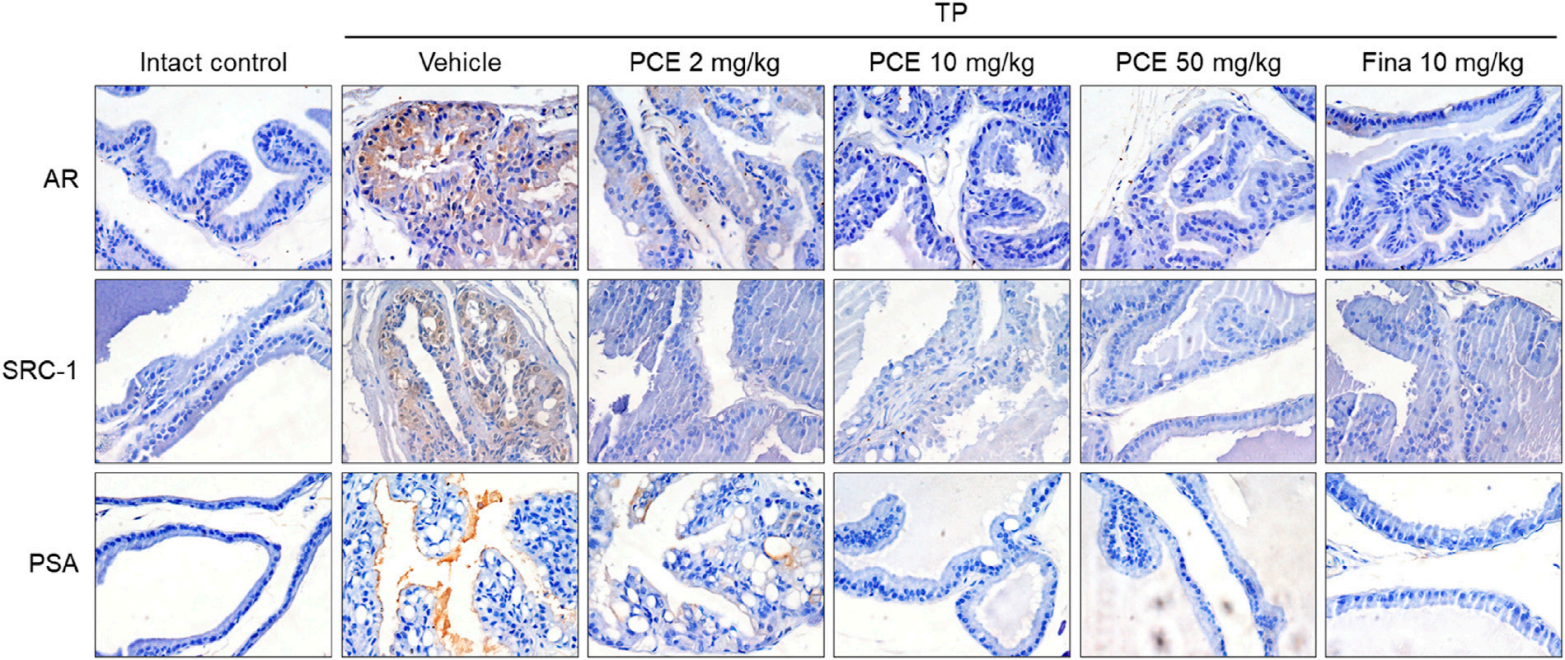
Effect of PCE on AR signaling-related protein expression in rats with TP-induced BPH. Immunohistochemical analyses were performed using anti-AR, anti-SRC-1, and anti-PSA antibodies. Representative images are presented in the original magnification ×60. Fina, finasteride; TP, testosterone propionate.

Immunohistochemical analyses were performed to determine the levels of ARs and SRC-1 in the tissue samples of TP-induced rats and whether PCE could affect the AR-mediated signaling cascade. The expression levels of both proteins were dramatically upregulated in the tissue specimens from the TP-induced control group compared to the normal group, but the levels were effectively downregulated in the PCE- and finasteride-treated groups. The expression levels of PSA, one of the downstream target genes of AR/SRC-1 signaling, showed a consistent expression pattern of ARs and SRC-1 (Figure 3).

**FIGURE 4:**
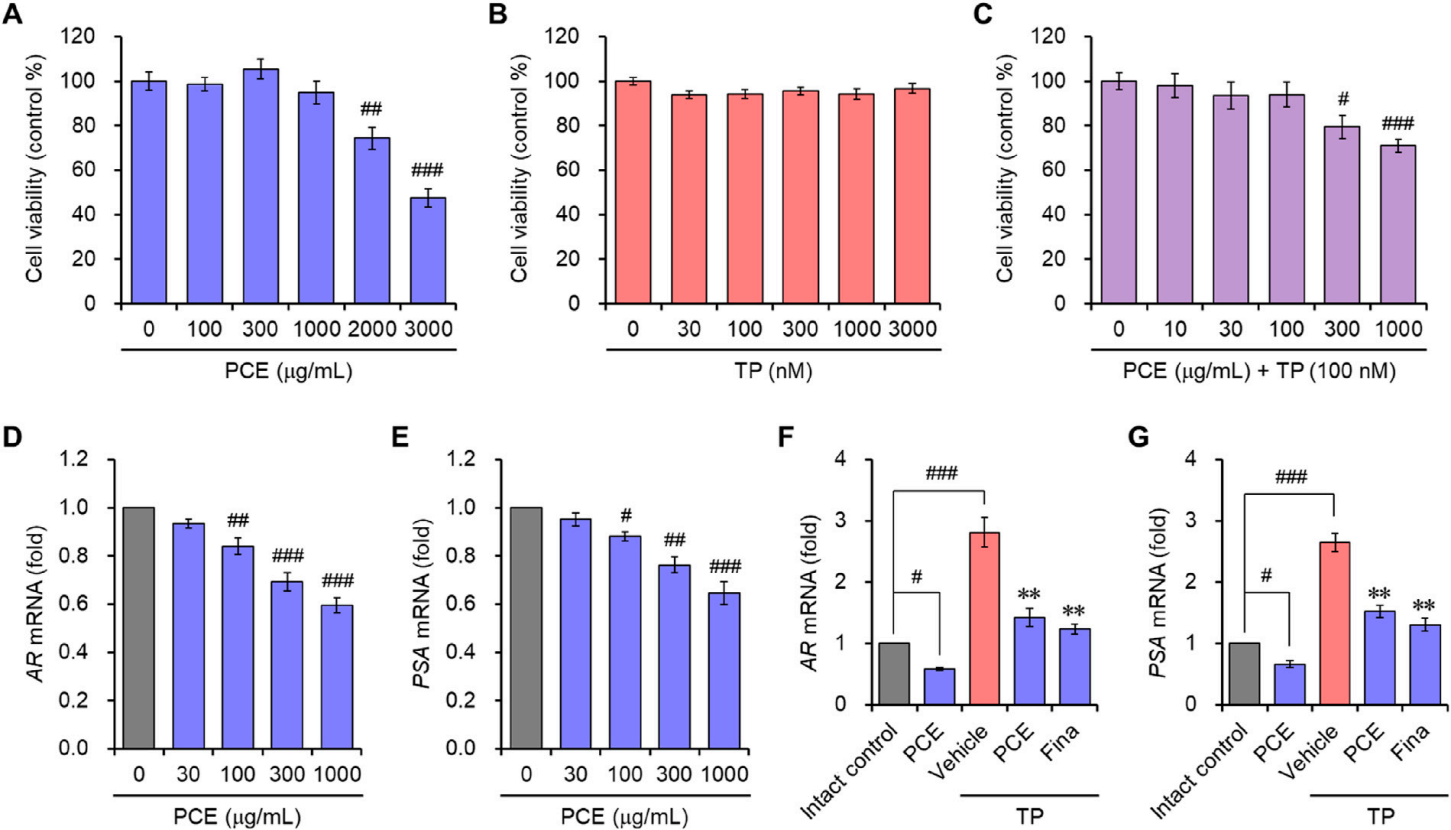
Cell viability and the mRNA expression of AR and PSA in LNCaP cells. **(A–C)** Cells were incubated with various concentrations of **(A)** PCE, **(B)** TP, or **(C)** various concentrations with PCE in the presence of 100 nM TP for 24 h, and their viability was determined. The results are expressed a as percentages of the control and are presented as means ± SEMs (*n* = 3). ^#^ *p* < 0.05, ^##^ *p* < 0.005, and ^###^ *p* < 0.0005 versus the vehicle-treated control group. TP, testosterone propionate. **(D–G)** Cells were incubated with the indicated concentrations of PCE, or either 1,000 μg/mL PCE or 10 μg/mL finasteride for 24 h in the presence or absence of 100 nM TP. Total RNA was extracted, and the mRNA expression of **(D, F)** AR and **(E, G)** PSA was analyzed using quantitative real-time PCR. The results are expressed as relative values compared to the vehicle-treated control and are presented as means ± SEMs (*n* = 3). ^#^ *p* < 0.05, ^##^ *p* < 0.005, and ^###^ *p* < 0.0005 versus the vehicle-treated control group. **p < 0.005 versus the TP-treated group. Fina, finasteride; TP, testosterone propionate.

The effects of PCE on AR-mediated signaling were further demonstrated in the human prostate cell line LNCaP, which is an androgen- and estrogen-sensitive adenocarcinoma. Cell viability was assessed to determine the appropriate experimental concentrations of PCE and TP before examining the effect of PCE on AR-mediated signaling. The results showed that cytotoxic activity was not observed up to 1,000 μg/mL PCE or 3,000 nM TP when cells were incubated for 24 h (Figures 4A, B). Thus, concentrations of PCE and TP up to 1,000 μg/mL and 100 nM were used in the *in vitro* experiments to exclude their cytotoxic effects.

*In vitro* AR-mediated signaling was determined by evaluating the mRNA levels of ARs and PSA. To determine their expression, LNCaP cells were incubated with PCE in the presence or absence of TP for 24 h. PCE treatment suppressed the mRNA levels of ARs and PSA in a concentration-dependent manner (Figures 4D, E). Although the mRNA levels were further elevated by the addition of TP in the cells, these levels were significantly suppressed by treatment with PCE or finasteride, and their effects were similar (Figures 4F, G). The *in vitro* and *in vivo* results imply that PCE inhibits the AR-mediated signaling pathway.

### 3.4 PCE increases apoptotic cell death

**FIGURE 5:**
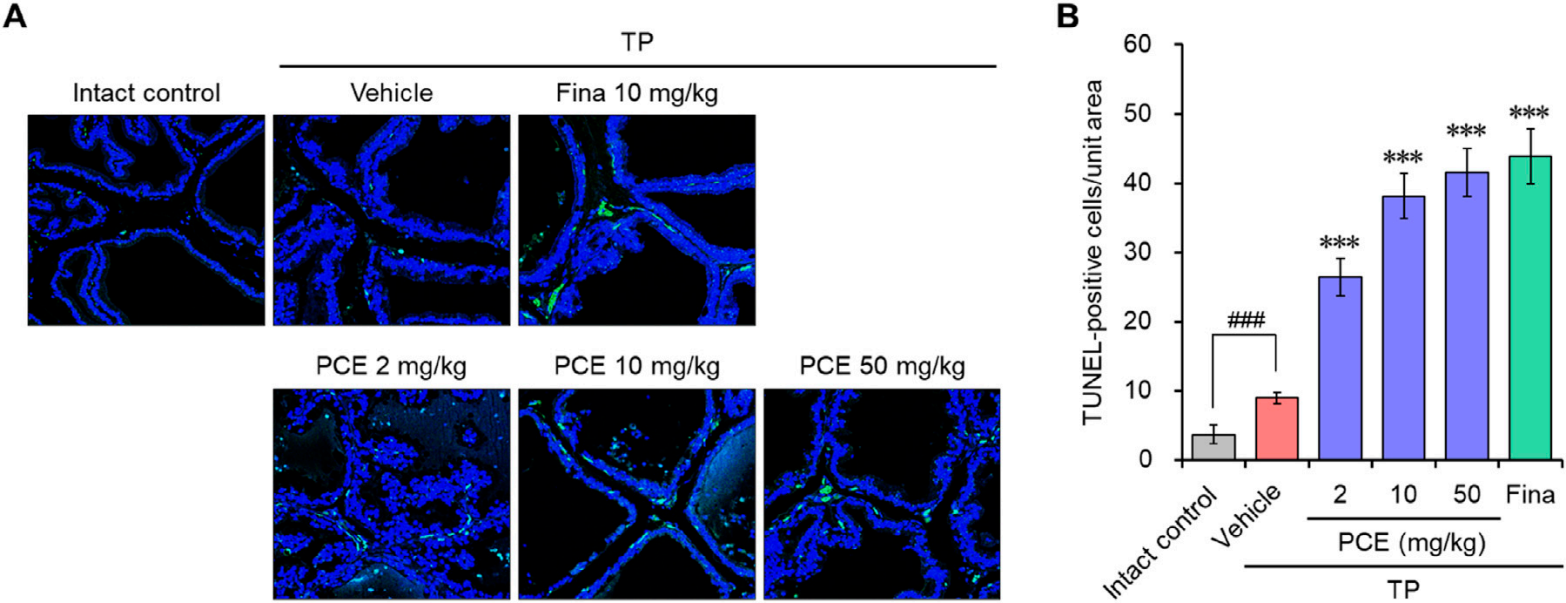
Effects of PCE on apoptotic cell death in rats with TP-induced BPH. TUNEL staining was carried out to analyze apoptotic cell death in the BPH tissues. **(A)** Representative images and **(B)** the TUNEL-positive cells in the tissue sections are shown. The results are represented as means ± SEMs (*n* = 7). ^###^ *p* < 0.0005 versus the normal control group. ***p < 0.0005 versus the TP-induced BPH group. Fina, finasteride; TP, testosterone propionate.

DHT binding to ARs directly triggers an increase in prostate volume and BPH development by inducing hyperproliferation of epithelial and stromal cells in the prostate. Therefore, regulating the balance between cell proliferation and apoptotic cell death is an important for treating BPH (Minutoli et al., 2016). Since we observed that PCE reduced the serum DHT levels by inhibiting the expression of SRD5A2 and AR-mediated signaling cascade, TUNEL staining was performed in the prostate tissues to quantify apoptotic cell death. TUNEL-positive cells were rarely detected in the tissue sections from the normal and TP-induced control groups. The TUNEL staining and elevated hyperplasia of the prostate epithelium in the TP-induced control group indicated that hyperproliferation dominated apoptosis in the BPH tissues of the TP-injected rats. However, the number of apoptotic cells was increased by administration of PCE or finasteride, and the effects at 10 and 50 mg/kg PCE were comparable to that of finasteride (Figure 5). Specifically, although PCE did not affect the viability of LNCaP cells up to 1,000 μg/mL, PCE reduced cell viability from 300 μg/mL when incubated with TP (Figure 4C).

**FIGURE 6:**
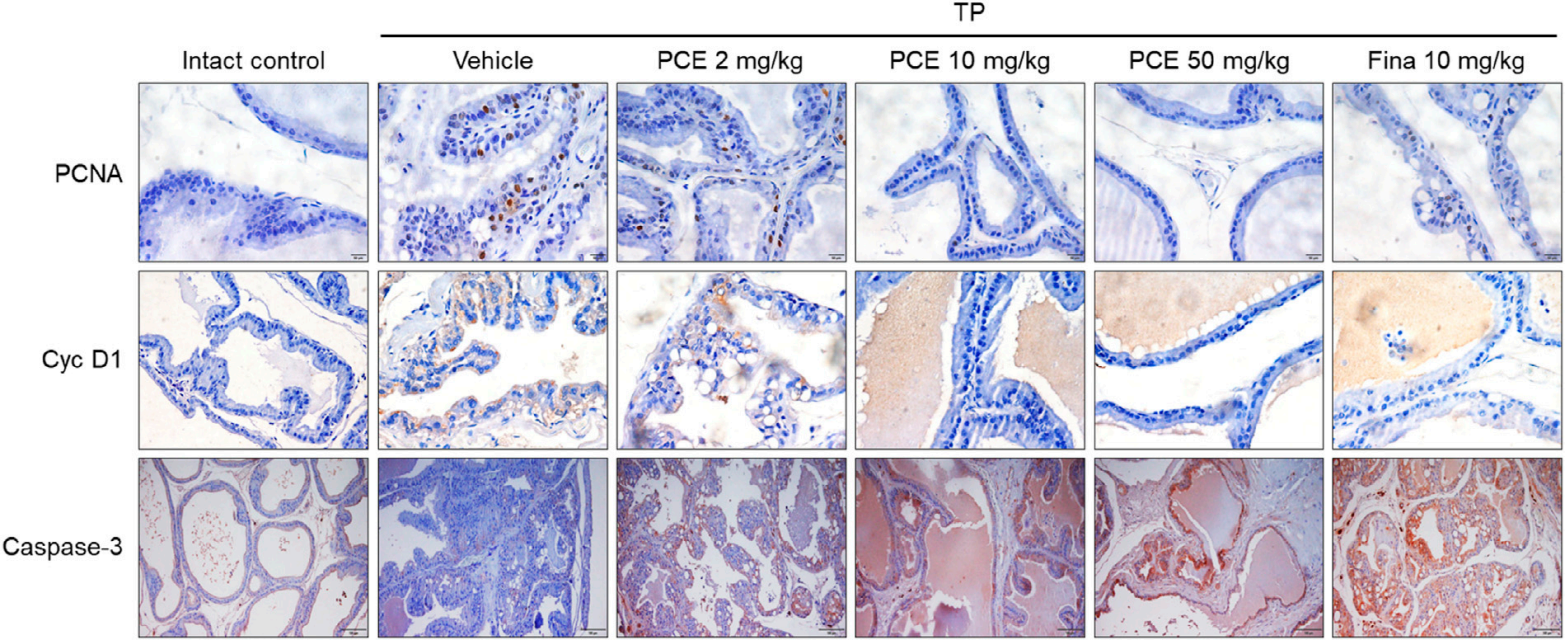
Effect of PCE on cell proliferation and apoptotic signaling-related protein expression in rats with TP-induced BPH. Immunohistochemical analyses were performed using anti-PCNA, anti-cyclin D1, and anti-caspase-3 antibodies. Representative images are presented at the original magnification (×20 for Caspase-3 or 60x for PCNA and Cyc D1). Fina, finasteride; TP, testosterone propionate.

In order to further confirm the effect of PCE on the regulation of cell proliferation and apoptosis, the expression of the cell proliferation markers proliferating cell nuclear antigen (PCNA) and cyclin D1 and the apoptotic cell marker caspase-3 was determined via immunohistochemical analyses. PCNA and cyclin D1 were highly upregulated in the tissues from the TP-induced control group compared to the normal group, but their expression was significantly reduced by administration of PCE or finasteride. On the other hand, the patterns of caspase-3 expression showed the opposite trend and was markedly increased in the PCE- and finasteride-treated groups (Figure 6). In particular, the effects of 10 and 50 mg/kg PCE on the cell proliferation markers and apoptotic marker were statistically significant and comparable to those of finasteride. These results clearly indicate that PCE regulates the imbalance between cell proliferation and apoptotic cell death. It thereby maintains the homeostasis of cell proliferation and death in TP-induced BPH rats.

### 3.5 PCE exhibits anti-inflammatory activity

Prostatic inflammation and various types of proinflammatory mediators are observed in BPH tissues, and these mediators, which are produced in prostate epithelial cells and stromal fibroblasts, are involved in hyperproliferation of epithelial and stromal cells in the prostate (Vela Navarrete et al., 2003; Roehrborn, 2008; Robert et al., 2009; Tsunemori and Sugimoto, 2021). This evidence suggests that prostatic inflammation acts as an important regulator of the pathogenesis of BPH. Therefore, immunohistochemical analyses of proinflammatory mediators were performed to investigate whether the improvement of BPH symptoms with PCE is associated with anti-inflammatory activity.

**FIGURE 7:**
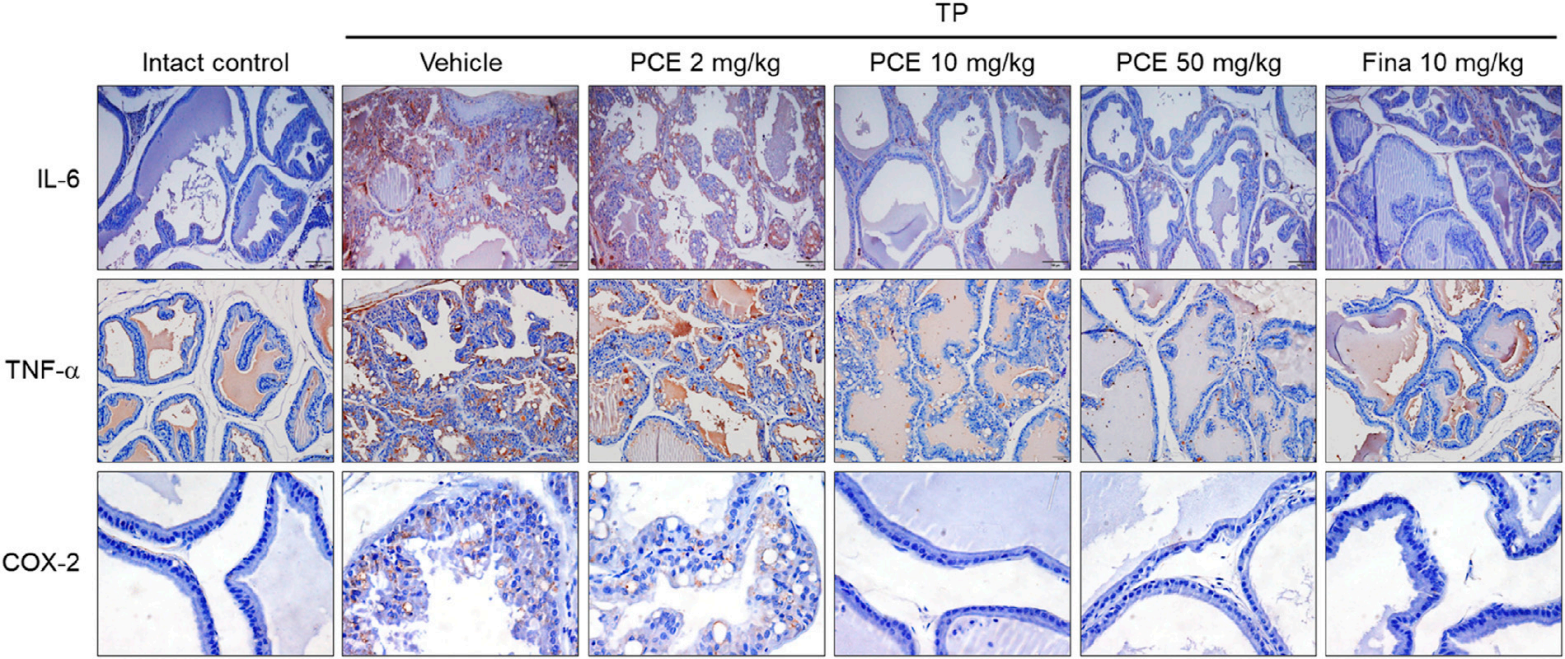
Effect of PCE on inflammatory signaling in rats with TP-induced BPH. Immunohistochemical analyses were performed using anti-IL-6, anti-TNF-α, and anti-COX-2 antibodies in rats with TP-induced BPH. Representative images are presented at the original magnification (×20 for IL-6 and TNF-α or 60x for COX-2). Fina, finasteride; TP, testosterone propionate.

**FIGURE 8:**
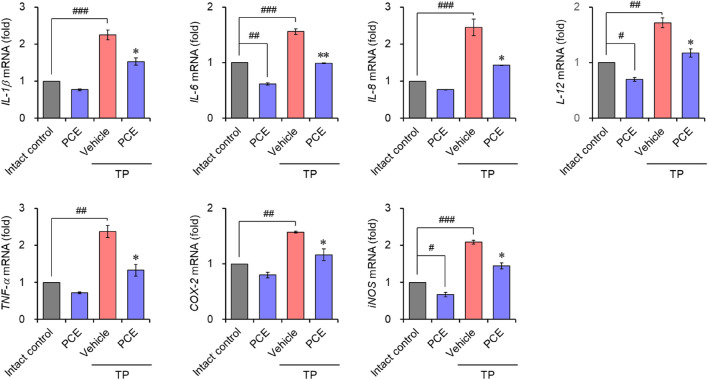
Effect of PCE on inflammatory signaling in LNCaP cells. Cells were incubated with 1,000 μg/mL PCE or 10 μg/mL finasteride for 24 h in the presence or absence of 100 nM TP. Total RNA was extracted, and the mRNA expression of inflammatory mediators was analyzed using quantitative real-time PCR. The results are expressed as relative values compared to the vehicle-treated control and are presented as means ± SEMs (*n* = 3). ^#^ *p* < 0.05, ^##^ *p* < 0.005, and ^###^ *p* < 0.0005 versus the vehicle-treated control group. *p < 0.05 and **p < 0.005 versus the TP-treated group.

Injection of TP resulted in markedly elevated levels of proinflammatory mediators, including interleukin-6 (IL-6), tumor necrosis factor-alpha (TNF-α), and cyclooxygenase-2 (COX-2), compared to the normal group, but their expression was significantly decreased by administration of PCE or finasteride. Administration of 10 or 50 mg/kg PCE almost restored the levels to those of the normal group, and the results were comparable to or stronger than those of finasteride (Figure 7). Similar results were observed via qPCR analyses in LNCaP cells after incubation with PCE in the presence or absence of TP for 24 h. The mRNA levels of proinflammatory molecules, including IL-1β, IL-6, IL-8, IL-12, TNF-α, COX-2, and inducible nitric oxide synthase (iNOS), were significantly decreased by PCE treatment, despite being increased by TP induction (Figure 8). These findings indicate that PCE exhibits anti-inflammatory activity in BPH rats, and this activity may play an important role in improving BPH symptoms.

### 3.6 PCE regulates the NF-κB and Nrf-2 axis

**FIGURE 9:**
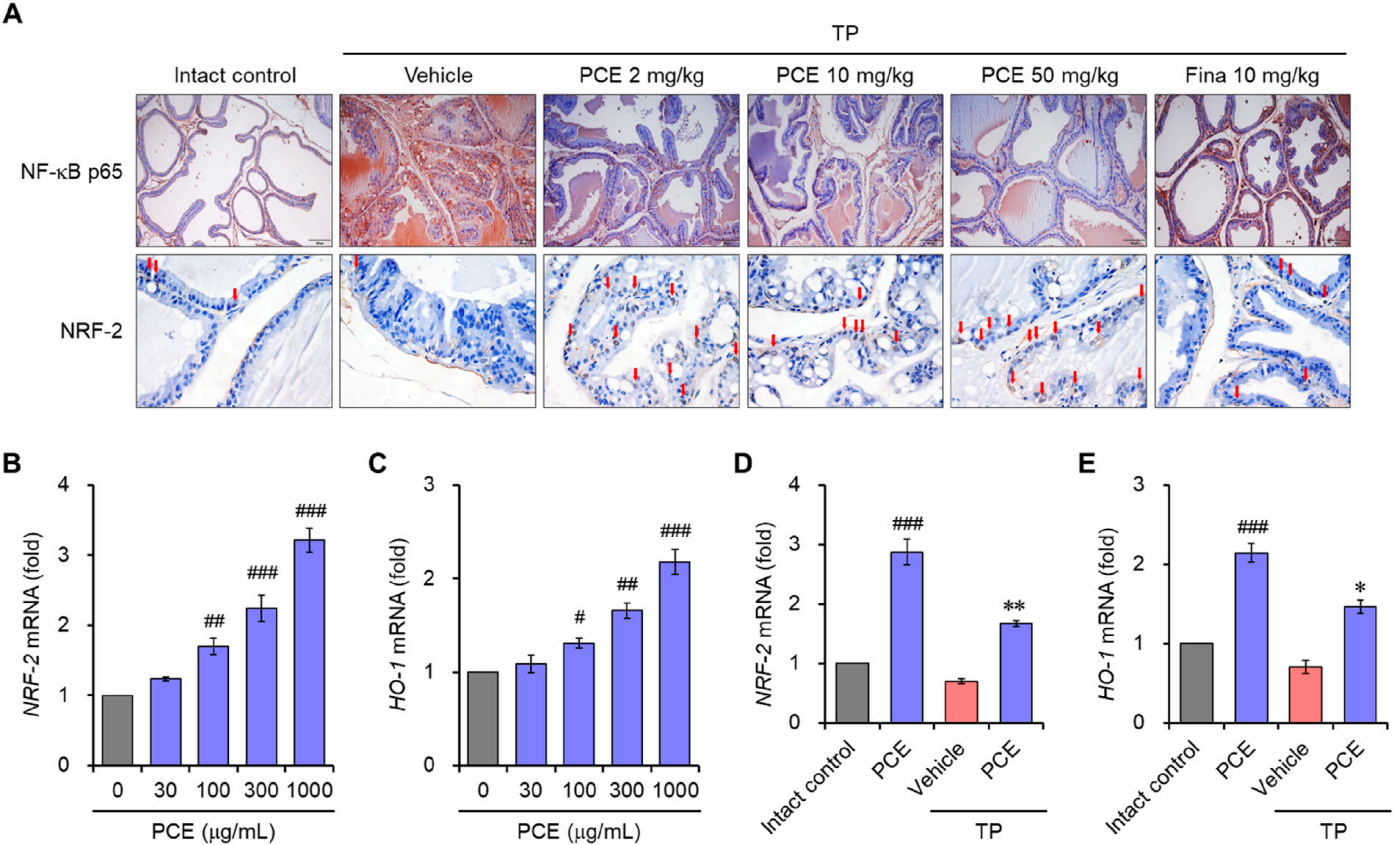
Effect of PCE on NF-κB and Nrf2 signaling. **(A)** Immunohistochemical analyses were performed using anti- NF-κB and anti-Nrf2 antibodies in rats with TP-induced BPH. Representative images are presented at the original magnification (×20 for NF-κB or 60x for NRF-2). Scale bar = 100 μm. Fina, finasteride; TP, testosterone propionate. Red arrows indicate NRF-2. **(B–E)** LNCaP cells were incubated with 1,000 μg/mL PCE or 10 μg/mL finasteride for 24 h in the presence or absence of 100 nM TP. Total RNA was extracted, and the mRNA expression of **(B, D)** Nrf2 and **(C, E)** HO-1 was analyzed using quantitative real-time PCR. The results are expressed as relative values compared to the vehicle-treated control and presented as means ± SEMs (*n* = 3). ^#^ *p* < 0.05, ^##^ *p* < 0.005, and ^###^ *p* < 0.0005 versus the vehicle-treated control group. *p < 0.05 and **p < 0.005 versus the TP-treated group.

The nuclear factor-kappa B (NF-κB) and nuclear factor erythroid 2-related factor 2 (Nrf-2)-mediated oxidative stress pathway plays an important role in the inflammatory response and apoptosis via macrophage activation in BPH (Li et al., 2019; Song et al., 2023). Therefore, to understand the modes of the action mechanism of PCE, the effects of PCE on NF-κB and Nrf-2 were investigated. Immunohistochemical analyses showed that the protein levels of NF-κB were dramatically elevated in the TP-induced control group compared to the normal group, but the levels were effectively decreased by administration of PCE or finasteride. On the contrary, the levels of Nrf-2 were reduced in the TP-induced control group compared to the normal group, and the protein levels were restored by administration of PCE or finasteride (Figure 9A).

We further investigated the regulation of Nrf-2 and heme oxygenase-1 (HO-1) by PCE in LNCaP cells. Prostatic inflammation can induce oxidative stress by producing reactive oxygen species (ROS) and reactive nitrogen species (RNS), and HO-1 is a Nrf-2-regulated gene that maintains homeostasis during inflammation-mediated oxidative stress (Song et al., 2023). Treatment with PCE upregulated the mRNA levels of Nrf-2 and HO-1 in a concentration-dependent manner (Figures 9B, C). In addition, their levels were significantly increased by treatment with PCE compared to TP, even though the mRNA levels were suppressed by TP stimulation (Figures 9D, E). These results suggest that PCE exhibits anti-inflammatory activity, and this activity may be involved in the regulation of the NF-κB and Nrf-2 axis.

## 4 Discussion

BPH is one of the most common urological diseases in the male population, and it includes BOO, lower urinary tract symptoms (LUTSs), and benign prostatic enlargement (BPE). The prevalence of BPH increases in middle-aged and older males, resulting in a significant reduction in quality of life (Ng and Baradhi, 2022). BPH symptoms are closely related to an enlarged prostate due to hyperproliferation of the prostate stromal cells, which are composed of a multitude of different cell populations, including endothelial cells, fibroblasts, smooth muscle cells, and immune cells (Pederzoli et al., 2023). Thus, regulation of stromal cell hyperproliferation is a main therapeutic target for BPH despite the unclear identification of the underlying molecular mechanisms for this progression. In fact, many natural and synthetic molecules have been reported to improve BPH symptoms by decreasing prostate enlargement (Eid and Abdel-Naim, 2020; Fu et al., 2022). In this study, we observed that PCE reduced prostate enlargement, the epithelial thickness of prostate tissues, and the prostate index without affecting body weight in TP-induced BPH rats, suggesting that PCE is a novel substance that may be beneficial for BPH treatment in humans.

In general, purple corn contains large amounts of anthocyanins, phenolic acids, and flavonoids, which have a variety of biological functions and are therefore known to be beneficial. As previously reported, we investigated the biological effects of PCE prepared from a new purple corn extract developed and registered by the Gangwon Agricultural Research and Extension Services. It has been reported that corn husks and cobs contain a variety of nutritional components, including large amounts of polyphenols and flavonoids, and among them, the total anthocyanin contents are very high (Lee et al., 2016; Lee et al., 2018; Lee et al., 2020; Kim et al., 2021). The six major components of anthocyanins were identified in the PCE, with C3G being the major component (Kim et al., 2022; Lee et al., 2023). In addition, extracts have beneficial biological roles such as anti-oxidant, anti-diabetic, anti-obesity, and anti-inflammatory effects, and improving dry eye symptoms (Lee et al., 2016; Lee et al., 2018; Lee et al., 2020; Kim et al., 2021; Kim et al., 2022; Lee et al., 2023). Along with these effects, many pharmacological activities have also been reported in purple corn (Lao et al., 2017; Cristianini and Guillén Sánchez, 2020; Kim et al., 2023). Furthermore, the toxicity and safety of purple corn have been evaluated through acute and 90-day subchronic oral toxicity studies in rats (Zhou et al., 2007; Nabae et al., 2008). The no-observed-adverse-effect level (NOAEL) values were determined to be 3,542 and 3,849 mg/kg body weight/day in male and female rats, respectively (Nabae et al., 2008). We also observed no toxic activity of PCE in acute and subchronic oral toxicity studies in rats or beagle dogs, with NOAEL values above 2,000 mg/kg. In addition, PCE showed no genotoxicity, including bacterial reverse mutation assay, *in vitro* mammalian chromosomal aberration assay, and *in vivo* mammalian erythrocyte micronuclear assay (data not shown). Therefore, PCE was found to be safe enough to have no limitation on the recommended dosage for consumption. This study further reported the anti-BPH effect of PCE through anti-inflammatory activity via regulation of the NF-κB and Nrf-2 axis. Furthermore, PCE decreased DHT production by inhibiting SRD5A2 expression, thereby inhibiting the conversion of testosterone to DHT.

Although the molecular mechanisms of BPH are still unclear, high levels of DHT are known to play a key role in the progression of BPH due to its higher affinity for ARs compared to testosterone, and the binding of DHT to the ARs initiates the AR signaling cascade (Carson and Rittmaster, 2003; Tong and Zhou, 2020). The functional AR signaling cascade consists of the synthesis of testosterone in the testes and adrenal glands, conversion of testosterone to DHT by 5α-reductase synthesized in prostate stromal cells, binding of DHT to its specific target receptor (AR), translocation of the AR-DHT complex to the nucleus, and binding of the AR-DHT complex to the DNA in a specific region with a co-regulator such as SRC-1 (Michmerhuizen et al., 2020). In fact, the AR signaling axis is the major therapeutic target for BPH and prostate cancer. To date, several substances have been developed to target the AR signaling axis, and some of them are used clinically for BPH and prostate cancer (Roehrborn et al., 1999; Roehrborn et al., 2002; Roehrborn et al., 2003; Saranyutanon et al., 2019). PCE effectively decreased serum DHT levels by suppressing 5α-reductase type 2, resulting in an increase in serum testosterone, and these results were similar to the mode of action of finasteride and dutasteride (Roehrborn et al., 2003; Hong et al., 2010). Two types of 5α-reductase have been identified: type 2 is predominantly expressed in the prostate, while type 1 is mainly expressed in the liver and skin (Steers, 2001), indicating that the type 2 enzyme plays a more important role in the progression of prostate hyperproliferation. In addition, PCE also decreased the expression of ARs and SRC-1, thereby decreasing the expression of PSA, one of the downstream target genes on the AR-DHT axis associated with BPH progression due to hyperproliferation of stromal and epithelial cells. These results clearly explain the modes of action by which PCE improves BPH. In addition, the inhibitory activities of PCE at 10 and 50 mg/kg were similar to that of 10 mg/kg finasteride. The inhibitory activity of PCE on 5α-reductase type 2 plays an important role in ameliorating BHP symptoms. This inhibitory activity consequently reduces DHT production while increasing testosterone concentration, thereby inhibiting the AR-mediated signaling pathway. Hence, our results imply that PCE may be a substance that can effectively improve BPH. In fact, finasteride and dutasteride are currently used in clinical trials to treat BPH by controlling hormonal imbalances. They are 5α-reductase inhibitors (5ARI) that inhibit the conversion of testosterone to DHT, which is identical to one of the action mechanisms of PCE. These agents cause a variety of serious adverse effects such as chest pain or discomfort, extreme fatigue, depression, irregular breathing, trouble breathing, swelling in the face, fingers, feet, or low legs, weight gain, anaphylaxis, and sexual problems, including decreased erection and ejaculation (Hirshburg et al., 2016; Lee et al., 2019). However, PCE is prepared from purple corn, a natural plant that has long been cultivated for food crop, and with little known side effects.

Cell proliferation and cell death are tightly controlled by the cell cycle, and a wide range of factors are involved. Several types of cells are present in the prostate, and the homeostasis between the proliferation and death of the component cells is delicately regulated by androgen signaling, which plays an important role in the development of the prostate. However, an imbalance between cell proliferation and cell death, especially, when proliferation prevails over cell death, results in prostate enlargement. Androgen signaling is reactivated by many stroma-derived growth factors during aging, which leads to BPH pathogenesis by inducing epithelial and fibroblastic proliferation and differentiation (Kassen et al., 1996; Planz et al., 1998; Niu et al., 2001), suggesting that inhibiting proliferation and promoting cell death, specifically apoptosis, in the prostate could be a valuable strategy for ameliorating BPH. We observed that PCE increased TUNEL-positive cells in the prostate, which implies that apoptotic cell death is promoted by PCE administration in BPH rats. According to the immunohistochemical analysis of the prostate tissues, the expression of the cell proliferation markers PCNA and cyclin D1 was decreased but the expression of the apoptotic cell marker caspase-3 was increased by PCE treatment in the BPH-induced rats. The results suggest that PCE decreases cell proliferation and increases apoptotic cell death. Increased apoptosis due to PCE was also demonstrated in our previous reports (Kim et al., 2022). PCE regulated PI3K/AKT signaling and thereby suppressed the expression of the anti-apoptotic markers Bcl-2 and BCL-xL and increased the expression of the pro-apoptotic marker Bax in the DHT-stimulated normal human prostate stromal cell line WPMY-1. These results indicate that PCE’s promotion of apoptosis while inhibiting cell proliferation plays an important role in improving the pathogenesis of BPH, since hyperproliferation of cells comprising the prostate is essential for prostate enlargement.

Along with androgen signaling, several factors are also implicated in the progression of BPH via androgen-independent pathways (De Nunzio et al., 2020; Hata et al., 2023). Prostatic inflammation is one of the risk factors for increased prostate volume. The volume depends on the grade of the inflammation because inflammatory cells are recruited to the prostate and the phenotypes of the cells in the prostate are affected by cytokines released from inflammatory cells (Begley et al., 2008; Roehrborn, 2008; Madersbacher et al., 2019; Tsunemori and Sugimoto, 2021). In addition, prostatic inflammation and inflammatory mediators promote cell proliferation and cause hyperplasia in the prostate during the healing process by inducing damage to cells and DNA, promoting cell replacement, and creating the tissue microenvironment (Cao et al., 2022; Hata et al., 2023; Naiyila et al., 2023). Furthermore, prostate tissue remodeling occurs via chronic inflammation in BPH through the wound healing process (Hata et al., 2023). We previously reported the anti-inflammatory activity of PCE in a desiccation stress-induced model (Lee et al., 2023). In that model, PCE decreased the mRNA levels of proinflammatory mediators in human retinal pigment epithelial cells and primary human corneal epithelial cells. PCE consistently exhibited anti-inflammatory activity in the testosterone-induced BPH rat model and LNCaP human prostate cancer cells. The reduced expression of anti-inflammatory molecules implies that PCE concurrently improves BPH through the regulation of androgen-independent and androgen-dependent signaling pathways.

Proinflammatory molecules can activate a wide range of signaling networks and are involved in cell proliferation and growth. Among these networks, the NF-κB pathway is representative of inflammatory signaling. Together with AR signaling, it regulates the progression of BPH by regulating the transcription of genes involved in inflammation and cell proliferation (Austin et al., 2016; Hata et al., 2023; Naiyila et al., 2023). Inflammation is induced by bacterial and viral infections in the prostate, and proinflammatory mediators produced in the stromal cells during BPH lead to prostatic growth by increasing cellular hyperproliferation. The autoimmune responses against self-antigens released from injured tissues play an important role in cellular hyperproliferation. In addition to the NF-κB and AR signaling pathways, oxidative stress is also considered an important trigger that produces reactive oxygen species (ROS), leading to the development and progression of BPH (Hata et al., 2023; Naiyila et al., 2023). Reactive intermediates, including free radicals and peroxides, are produced during metabolic processes, and high levels of these intermediates results in cell and tissue damage and ultimately disrupt cellular functions by altering the intracellular signaling pathways. Controlling oxidative stress can reduce inflammation, and oxidative stress can be regulated by the body’s anti-oxidant defense and detoxification systems (Forman and Zhang, 2021), indicating the importance of regulating oxidative stress for maintaining health. Unfortunately, however, the prevalence of oxidative stress increases with age because the pro-oxidant function is preferred compared to the anti-oxidant function. These results suggest that reducing inflammation and increasing the anti-oxidant ability are valuable therapeutic strategies for improving BPH. We found that PCE decreased the expression of NF-κB and increased the expression of Nrf-2 in the rat BPH tissue samples as well as the LNCaP prostate carcinoma cells. In addition, PCE upregulated the mRNA expression of HO-1. Indeed, Nrf-2 is a regulator of inflammation that is activated by androgens and plays an important role in controlling cell proliferation and apoptosis in BPH (Li et al., 2019; Song et al., 2023). HO-1 is a downstream target of Nrf-2. Its promoter activity is synergistically activated by Nrf-2 and breast cancer susceptibility protein 1 (BRCA1), and the BRCA1-Nrf-2/HO-1 complex is responsible for maintaining cellular homeostasis (Labanca et al., 2015; Udensi and Tchounwou, 2016). In general, antioxidants are known to prevent and alleviate chronic inflammatory diseases by reducing oxidative stress and secretion of inflammatory molecules. PCE contains many biological active substances such as anthocyanins, flavonoids, and polyphenols, and their antioxidant activity can lead to anti-inflammatory activity. According to the roles of the NF-κB and Nrf-2/HO-1 signaling pathways, PCE exerts anti-inflammatory effects and improves oxidative stress, thereby ameliorating BPH by regulating inflammatory and oxidative stress-mediated prostatic inflammation and cell proliferation in the prostate.

## 5 Conclusion

This study was demonstrated that PCE improved BPH symptoms in TP-induced rats. This effect was mediated by the inhibition of the 5α-reductase type 2 and the AR-mediated signaling pathway, as well as anti-inflammatory activity via the regulation of the NF-κB/Nrf2 axis. It decreased serum DHT levels and regulated the homeostasis of hyperproliferation and death in cells surrounding in the prostate. Our findings thus indicate that PCE is beneficial to human health and can safely be used as a functional food material for improving BPH symptoms.

## Data Availability

The original contributions presented in the study are included in the article/supplementary material, further inquiries can be directed to the corresponding author.
